# Period poverty: a scoping review

**DOI:** 10.1590/0034-7167-2024-0567

**Published:** 2025-06-20

**Authors:** Mayara Cristina de Paula, Bruna Borlina Monteiro, Ludmila de Oliveira Ruela, Mônica Maria de Jesus Silva

**Affiliations:** I Universidade de São Paulo. Ribeirão Preto, São Paulo, Brazil

**Keywords:** Nursing, Menstruation, Poverty, Menstrual Hygiene Products, Reproductive Health., Enfermería, Menstruación, Pobreza, Productos para la Higiene Menstrual, Salud Reproductiva.

## Abstract

**Objectives:**

to map evidence, perspectives and gaps on period poverty.

**Methods:**

a scoping review, carried out in May 2022 in the MEDLINE via PubMed, Scopus, LILACS, CINAHL, EMBASE and Web of Science databases, according to the JBI recommendations.

**Results:**

the final sample consisted of 23 studies. Four predominant factors in relation to period poverty were observed: factors associated with period poverty; impacts of period poverty; menstrual hygiene management; and coping strategies.

**Conclusions:**

period poverty is a public health and human rights issue, especially for people in situations of socioeconomic vulnerability. Despite the growing international commitment to focus on promoting menstrual health, it still does not receive adequate emphasis. The importance of implementing strategies to address and overcome period poverty to promote menstrual health and achieve gender equality was highlighted.

## RESUMEN

**Objetivos:**

mapear la evidencia, perspectivas y brechas sobre la pobreza menstrual.

**Métodos:**

revisión del alcance, realizada en mayo de 2022 en las bases de datos MEDLINE vía PubMed, Scopus, LILACS, CINAHL, EMBASE y Web of Science, de acuerdo con las recomendaciones del JBI.

**Resultados:**

la muestra final estuvo compuesta por 23 estudios. Se observaron cuatro factores preponderantes en relación a la pobreza menstrual: factores asociados a la pobreza menstrual; impactos de la pobreza menstrual; gestión de la higiene menstrual; y estrategias de afrontamiento.

**Conclusiones:**

la pobreza menstrual es una cuestión de salud pública y derechos humanos, especialmente para personas en situación de vulnerabilidad socioeconómica. A pesar del creciente compromiso internacional de centrarse en la promoción de la salud menstrual, todavía no recibe el énfasis adecuado. Se destacó la importancia de implementar estrategias para combatir y superar la pobreza menstrual para promover la salud menstrual y lograr la igualdad de género.

## INTRODUCTION

Menstrual health and its importance have been historically neglected, mainly due to taboos and misconceptions surrounding menstruation and the impact of patriarchy on knowledge and health systems around the world^(1)^.

Menstrual health needs to be understood as a tool for promoting the health of people who menstruate, and is linked to experiences related to the menstrual cycle, which includes combating taboos, misinformation, lack of data, stigma and discrimination related to menstruation^(2)^.

It should be noted that, in this study, the term “people who menstruate” was used so as not to exclude people who have a menstrual cycle but do not identify as women, and thus include women, transgender men and non-binary people.

Menstrual health involves meeting appropriate menstrual hygiene needs by providing menstruators with access to accurate information about menstruation; clean menstrual products that can be changed privately as often as needed; sanitary materials, soap and water for washing and cleaning; a place for hygienic disposal of used products such as reusable pads; and clean and safe sanitary facilities for washing such as toilets^(1,3)^.

However, not all people who menstruate have access to these resources, and inequalities are present due to social and health inequities, which place them in a situation of period poverty, which refers to financial, social, cultural and political barriers to accessing menstrual products and education^(1)^. These barriers include financial resources to purchase menstrual hygiene supplies; transportation to travel to centers where supplies can be purchased in large quantities in an affordable manner; access to basic sanitation and privacy; protection and safety in accessing bathrooms; and access to information to manage the menstrual cycle^(3)^.

This condition of period poverty mainly affects people in vulnerable situations, since it is strongly supported by social inequalities and taboos that limit the natural process that characterizes menstruation^(4)^.

In this context, in 2014, the United Nations (UN) recognized women’s right to menstrual hygiene as a matter of public health and human rights. However, what should be a right is often a luxury. According to UN Women, 12.8% of the world’s female population lives in period poverty; 526 million people do not have access to a bathroom to menstruate with dignity; and 1.25 billion do not have access to a safe bathroom^(5)^.

In Brazil, around 30 million women menstruate and spend, over the course of their lives, between three and nine thousand *reais* on sanitary pads, and 1.24 million girls do not have access to toilet paper in the bathrooms of their schools^(4,6)^.

This scenario of period poverty promotes negative experiences around menstruation such as: school and work absenteeism due to lack of menstrual hygiene supplies; loss of productivity during the menstrual period; absence from social activities; gender discrimination^(7)^; precariousness of basic conditions with consequent improvisation of inadequate materials, such as cloths, tissues and/or paper towels or the prolonged use of internal absorbents^(3)^; development of infections; and low quality of life related to health^(8,9)^.

These consequences show that overcoming period poverty in all its nuances is essential to promoting the health of people who menstruate and to achieving gender equality. To this end, dissemination of information, support, visibility in government agendas and democratization of access to menstrual supplies are necessary.

In this regard, research, practice and policies that address menstrual health management and overcoming poverty involve a growing number of actors, such as researchers, practitioners, policy makers, social entrepreneurs and civil society, who globally have been working to address the social, environmental and political factors that reinforce the challenges related to menstruation faced by people who menstruate in different contexts^(10)^.

However, despite the recent global attention given to period poverty as a public health and human rights issue, especially in resource-poor countries^(11,12)^, and the growing international commitment to focus on menstrual health promotion, it still does not receive adequate emphasis and much remains to be done.

Therefore, knowing the gaps and perspectives regarding period poverty is essential to outline strategies and implement actions to overcome it and to promote the health of people who menstruate.

## OBJECTIVES

To map evidence, perspectives and gaps on period poverty.

## METHODS

### Ethical aspects

Ethical assessment was not necessary, since the material used is in the public domain and does not involve human beings.

### Study design

This is a scoping review of the literature, in which the guidelines proposed by the JBI^(13)^ were followed. Additionally, the Preferred Reporting Items for Systematic Reviews and Meta-Analyses Extension for Scoping Reviews (PRISMA-ScR) was used to write the study^(14)^.

### Study period and place

The databases used were MEDLINE (via PubMed), CINAHL (via EBSCO), Web of Science, Scopus, LILACS and EMBASE.

The descriptors were selected from databases in March and April 2022. Data collection took place in May 2022.

### Inclusion and exclusion criteria

Studies that answered the research question, met the study objective, and had a thematic approach to period poverty were considered eligible. There were no language or time restrictions. Studies in the form of commentary, editorial, protocol/review, debate, case study, theoretical study/reflection, technical scientific report, critique, book/film analysis, or abstract of a scientific conference/event were excluded.

### Study protocol

To guide the review, the mnemonic combination PCC^(13)^ was used, consisting of Population (not applicable), Concept (menstruation) and Context (period poverty). It is important to note that the population was not restricted to women and girls, so as not to limit the studies in terms of gender and aiming to cover all people with a uterus who menstruate. Thus, the research question was formulated: what evidence on the situation of period poverty is available in the literature?

Subsequently, the search for descriptors representing the object of study began in MEDLINE (via PubMed) and CINAHL (via EBSCO), followed by a broader search, using the same keywords and search terms in the Web of Science, Scopus, LILACS and EMBASE databases.

Subsequently, the descriptors were identified in the Medical Subject Headings (MeSH), for descriptors in English, and in the Health Sciences Descriptors (DeCS), for descriptors in Portuguese and Spanish. The selected descriptors were “Menstruation”, “Menstrual Hygiene Products”, “Menstrual*”, “Poverty” and “Period Poverty” in English, Spanish and Portuguese. The search strategy was developed in conjunction with a librarian from a public university in the state of São Paulo, adding the Boolean operators AND and OR to the descriptors.

After determining the descriptors and creating the above strategy, searches were carried out in each database. Then, an external search was carried out on Google Scholar^®^, targeting gray literature, as recommended by the JBI^(13)^. Finally, the reference list of the main articles included was checked to retrieve relevant articles.

The search for studies was carried out by two reviewers independently and blindly. First, the titles, abstracts and descriptors were read. Then, full texts were read. At each stage, consensus was reached among the reviewers through discussion. In case of disagreement, a third reviewer could be added, which was not necessary.

To aid selection and management, studies were exported to Rayyan QCIR^®^ software^(15)^.

### Analysis of results

Data were extracted, organized and characterized in an adaptation of a form recommended by the JBI, containing publication data (year, authors and country of publication), study objectives, methodological characteristics (study population) and main results (outcomes, findings or contributions).

The extracted information was organized in a spreadsheet in Microsoft Office Excel^®^. Study characterization data were analyzed using simple descriptive statistics. Evidence on period poverty was interpreted through consensus reached by the researchers after extracting the results independently.

## RESULTS

**Figure 1:**
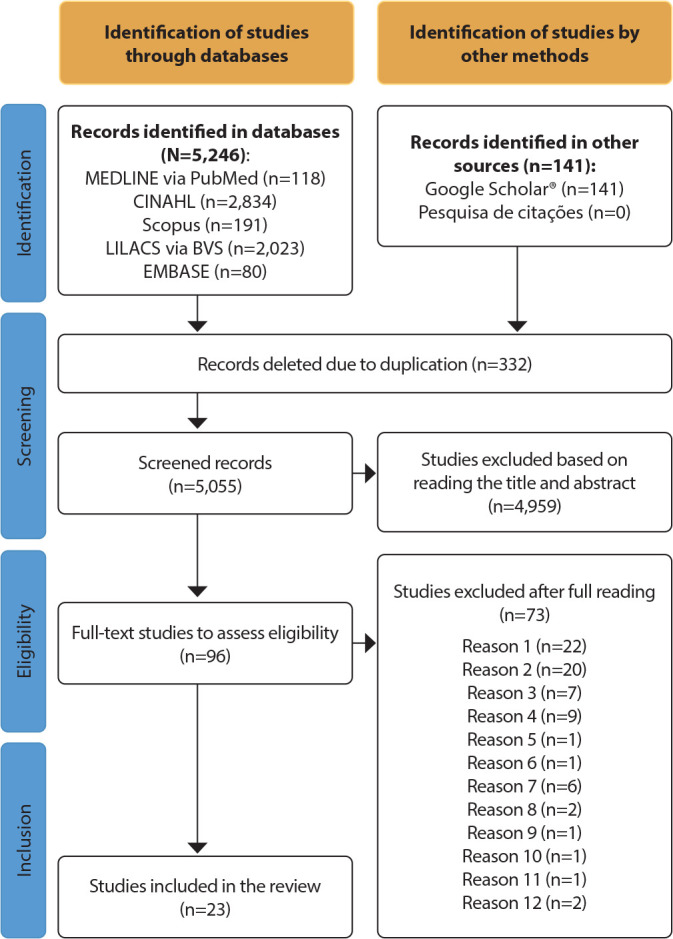
Study selection flowchart, Ribeirão Preto, São Paulo, Brazil, 2022

*Source: adapted from PRISMA-ScR^(13)^.*

*Legend: reason 1: does not answer the question; reason 2: not available in full text; reason 3: commentary/editorial; reason 4: protocol/review; reason 5: debate; reason 6: case study; reason 7: theoretical study/reflection; reason 8: dissertation; reason 9: technical scientific report; reason 10: criticism; reason 11: book analysis; reason 12: conference summary.*

Of the 5,246 studies identified in the initial search, 23 were included in the final sample. Figure 1 specifies the results of the analysis stages, following the PRISMA Flow Diagram model^(14)^.

Chart 1 presents a summary of results.

**Chart 1:** Characterization of studies regarding title, year, country, methodological design, publication journal and results on period poverty, Ribeirão Preto, São Paulo, Brazil, 2022

ID	Title/methodological design	Country/ year/journal	Results on period poverty
1^(16)^	Unmet Menstrual Hygiene Needs Among Low-Income Women Cross-sectional study	United States, 2019 Obstetrics & Gynecology	- Lack of access to menstrual hygiene products and safe use of sanitary facilities; - Challenges for low-income women similar to those experienced in countries with fewer resources than the United States of America.
2^(17)^	Adolescent Experience of Menstruation in Rural Kenya Qualitative study	Kenya, 2016 Nursing Research	- Sources of information about menstruation were school, family and colleagues; - Use of inadequate alternative materials due to lack of sanitary pads; - School absenteeism and impact on education; - Menstruation related to bad feelings.
3^(18)^	Menstrual health and school absenteeism among adolescent girls in Uganda (MENISCUS): a feasibility study Mixed-methods study	Uganda, 2018 BMC Women’s Health	- Source of information about menstruation were mothers; - Cultural norms unfavorable to menstruation; - Use of inadequate alternative materials due to lack of sanitary pads; - School absenteeism.
4^(19)^	Menstrual hygiene management amongst schoolgirls in the Rukungiri district of Uganda and the impact on their education: a cross-sectional study Mixed-methods study	Uganda, 2014 Pan African Medical Journal	- School absenteeism; - Lack of access to sanitary pads; - Poor conditions of school bathrooms for managing menstrual hygiene.
5^(20)^	Emotional and Psychosocial Aspects of Menstrual Poverty in Resource-Poor Settings: A Qualitative Study of the Experiences of Adolescent Girls in an Informal Settlement in Nairobi Qualitative study	Kenya, 2013 Health Care for Women International	- Source of information about menstruation was family, friends and teachers. Limited information; - Menstruation related to bad feelings; - Menstruation affected concentration and attendance in classes.
6^(21)^	Physical, Social, and Political Inequities Constraining Girls’ Menstrual Management at Schools in Informal Settlements of Nairobi, Kenya Qualitative study	Kenya, 2017 Journal Urban Health	- Lack of access to information about menstruation; - Free distribution of sanitary pads in schools; - Shame about menstrual blood.
7^(22)^	‘Period poverty’ in Stoke-on-Trent, UK: New insights into gendered poverty and the lived experiences of austerity Qualitative study	England, 2020 Journal of Poverty and Social Justice	- “Period poverty” related to low income; - Social exclusion due to menstruation; - Impacts on mental health.
8^(23)^	Menstrual hygiene - A salient hazard in rural schools: A case of Masvingo district of Zimbabwe Qualitative study	Zimbabwe, 2016 Jàmbá: Journal of Disaster Risk	- Religious and cultural beliefs unfavorable to menstruation; - Use of inadequate alternative materials due to lack of sanitary pads; - Limitations in menstrual hygiene management; - Absenteeism and poor school performance.
9^(24)^	Menstrual hygiene management practice among adolescent girls: an urban-rural comparative study in Rajshahi division, Bangladesh Cross-sectional study	India, 2022 BMC Women’s Health	- Source of information about menstruation were mothers, friends and teachers; - Difficulty in accessing sanitary pads; - Socioeconomic factors related to good menstrual hygiene practices.
10^(25)^	Effect of menstruation on girls and their schooling, and facilitators of menstrual hygiene management in schools: surveys in government schools in three states in India, 2015 Cross-sectional study	India, 2019 Journal of Global Health	- Religious and cultural beliefs unfavorable to menstruation; - Bad feelings related to menstruation; - Lack of conditions for menstrual hygiene management; - Facilitators to reduce school absenteeism during menstruation.
11^(26)^	Secrets, shame and discipline: School girls’ experiences of sanitation and menstrual hygiene management in a peri-urban community in Ghana Quantitative study	Ghana, 2019 Taylor & Francis	- Lack of conditions for managing menstrual hygiene; - Information given after menarche is more focused on sexual and reproductive health than on menstrual hygiene.
12^(27)^	Menstrual hygiene management among girls at a peri-urban senior high school in the Volta Region, Ghana Descriptive study	Ghana, 2020 African Journal of Midwifery and Women’s Health	- Menstruation seen as unhygienic; - Lack of information and access to sanitary pads; - School absenteeism; - Lack of conditions for managing menstrual hygiene in schools.
13^(28)^	Menstruation Management in United States Schools and Implications for Attendance, Academic Performance, and Health Qualitative study	United States, 2019 Women’s Reproductive Health	- Menstruation as something negative; - Lack of sanitary pads hinders concentration in class; - Distribution of free sanitary pads at school reduces the chance of absenteeism and impact on learning.
14^(29)^	Menstruation practice among school and out-of-school adolescent girls, Lao PDR Descriptive study	Laos, 2020 Global Health Action	- School and work absenteeism due to menstruation; - Menstruation was discussed more with mothers and little with fathers; - Factors related to good menstrual hygiene management practices.
15^(30)^	Menstrual hygiene management among women and adolescent girls in the aftermath of the earthquake in Nepal Mixed-methods study	Nepal, 2018 BMC Women’s Health	- Menstrual hygiene was considered the sixth greatest need after the earthquake in Nepal; - No women received donations of sanitary pads; - Poor conditions for managing menstrual hygiene; - Use of alternative materials not indicated in the absence of sanitary pads.
16^(31)^	Empowering Women: Teaching Ethiopian Girls to Make Reusable Sanitary Pads Cross-sectional study	Ethiopia, 2014 Clinical Scholars Review	- The project taught about empowerment, menstruation and how to sew reusable pads; - Talks about access to pads and shame about menstrual blood; - Poor bathroom conditions for menstrual hygiene management; - The use of reusable pads had a positive impact.
17^(32)^	Menstrual Hygiene among Adolescent Schoolgirls in Mansoura, Egypt Cross-sectional study	Egypt, 2005 Taylor & Francis	- Socioeconomic factors related to menstrual education and access to sanitary pads; - Lack of information about menstruation; - Sources of information were the media and mothers; - Cultural and religious beliefs unfavorable to menstruation.
18^(33)^	Period poverty and mental health implications among college-aged women in the United States Cross-sectional study	United States, 2021 BMC Women’s Health	- Factors related to access to sanitary pads; - Alternatives to deal with the lack of sanitary pads; - Association between having experienced period poverty and having symptoms of depression.
19^(34)^	Period poverty: menstrual health hygiene issues among adolescent and young Venezuelan migrant women at the northwestern border of Brazil Cross-sectional study	Brazil, 2021 Reproductive Health	- Among young Venezuelan migrants in Boa Vista, Brazil, there was a lack of access to adequate menstrual hygiene products, sanitary conditions and bathrooms; - Menstruation was seen as a taboo; - Lack of information about menstruation; - Menstruation was associated with negative feelings.
20^(35)^	Understanding period poverty: Socio-economic inequalities in menstrual hygiene management in eight low-and middle-income countries Cross-sectional study	South Africa, 2021 International Journal of Environmental Research and Public Health	- Lack of access to bathrooms with adequate conditions for menstrual hygiene management and sanitary pads; - Factors related to the lack of supplies for menstrual hygiene.
21^(36)^	Factors impacting on menstrual hygiene and their implications for health promotion Qualitative study	Zambia, 2018 Global Health Promotion	- Stigma and culture unfavorable to menstruation; - Lack of information about menstruation; - Absenteeism and school dropout; - Use of alternative materials due to lack of sanitary pads.
22^(37)^	Menstrual hygiene management in rural schools of Zambia: a descriptive study of knowledge, experiences and challenges faced by schoolgirls Quantitative study	Zambia, 2019 BMC Public Health	- Lack of information on menstrual hygiene management; - Negative experiences during the first menstruation; - Lack of access to sanitary pads; - School absenteeism; - Lack of sanitary conditions for menstrual hygiene management at school.
23^(38)^	*Desafíos de la menstruación en niñas y adolescentes de comunidades rurales del pacífico colombiano* Mixed-methods study	Colombia, 2017 *Rev. Salud Pública*	- Lack of conditions for menstrual hygiene management at school; - School absenteeism; - Lack of information about menstruation and menstruation as a negative experience; - There were no public policies for menstrual education.

The studies were published between 2005 and 2022, with one (4.3%) published in 2005^(32)^, one (4.3%) in 2013^(20)^, two (8.7%) in 2014^(19,31)^, two (8.7%) in 2016^(19,23)^, two (8.7%) in 2017^(21,38)^, three (13%) in 2018^(18,30,36)^, five (21.7%) in 2019^(16,18,26,28,37)^, three (13%) in 2020^(22,27,29)^, three (13%) in 2021^(33-35)^, and (4.3%) in 2022^(24)^.

The countries with the highest number of studies were United States^(16,28,33)^ and Kenya^(17,20,21)^, with three studies each (26%); India^(23,25)^, Uganda^(18,19)^, Gana^(26,27)^ and Zambia^(36,37)^, with two articles each (34,8%); followed by Brazil^(37)^, England^(22)^, Zimbabwe^(23)^, Laos^(29)^, Nepal^(30)^, Ethiopia^(31)^, Egypt^(32)^, South Africa^(35)^ and Colombia^(34)^, which presented a study each (38.7%).

As for methodology, nine (39.1%) were qualitative studies^(17,20-23,26,28,36,37)^, eight (34.7%) cross-sectional^(16,22,24,25,31,32,37)^, four (17.5%) mixed-methods study^(18-19,30,38)^, and two (8.7%) descriptive^(27,29)^.

The results highlighted four preponderant factors among the perspectives and gaps in relation to period poverty: factors associated with period poverty; impacts of period poverty; menstrual hygiene management; and coping strategies.

## DISCUSSION

As highlighted in the United Nations Children’s Fund report, period poverty is characterized as a transdisciplinary and multidimensional phenomenon experienced by people who menstruate due to the presence of economic, social, cultural and political barriers to the management of menstrual hygiene^(4)^, as observed in the studies in this review^(16-38)^.

Some socioeconomic factors are related to period poverty, such as low income^(16,17,22,35)^, education^(24,29,35)^ and housing in rural areas^(17,35)^. Given the economic factors, low-income menstruators were more likely to have difficulty accessing menstrual hygiene products and accessing basic sanitation in schools and homes^(16,17,36)^.

Furthermore, menstruating people living in rural areas are often forced to miss school^(17,36)^ due to the challenges they face in managing menstrual hygiene^(37)^, such as lack of access to sanitary pads and adequate infrastructure^(17)^.

In this regard, although period poverty is a global experience, the negative impact on quality of life and well-being may be more pronounced for people living in conditions of poverty and vulnerability^(4)^.

On the other hand, some factors are associated with ensuring good menstrual hygiene practices, such as living in an urban area, being part of a small family^(24)^, adequate parental education^(24,29)^, and conversations between mother and daughter^(29)^. Although there is still an association between access to menstrual hygiene products with low income and living in rural areas^(16,17,22,35)^, living in urban areas, when analyzed together with income, has an influence on greater or lesser period poverty, depending on the country in question^(35)^.

The lack of products for menstrual health management can affect even people who are not in poverty. This can be understood because sanitary pads are considered a superfluous product, leaving little or no income to be used to purchase supplies that help ensure menstrual dignity^(4)^.

Even though lack of financial resources is one of the main determinants of period poverty^(16,17,22,35)^, people who menstruate in low-income countries face menstrual hygiene challenges similar to those of people in countries with more resources^(16)^, highlighting that period poverty is a global phenomenon that needs to be addressed holistically.

Taboos, information restrictions and stigma surrounding menstruation, as well as a lack of adequate resources for menstrual hygiene management, negatively affect the ability of menstruating people to socialize and participate in school^(17-20,23,25,28,29,31,33)^. Social and physical barriers include inadequate toilets, water and disposal on school grounds^(18-21,23,25-27,29,34,35,37-38)^, lack of access to painkillers^(18,21,32)^, insufficient guidance and support to manage their menstrual periods^(18,20,21,32,37,38)^, and difficulty accessing menstrual products^(17-20,26,27,35)^.

The main reasons given for why menstruation keeps people who menstruate away from school include: pain^(18,19,37)^, as some did not take painkillers due to lack of knowledge or lack of money^(20)^; fear of humiliation due to exposure of menstrual blood^(17-20,25,37,38)^; lack of sanitary pads^(17-19,38)^; lack of privacy; and inadequate facilities for managing menstrual hygiene^(19,21,37)^.

The data presented demonstrate how children and adolescents who menstruate have their rights to quality education, health, including sexual and reproductive health violated when their rights to water, sanitation and hygiene are not guaranteed in the spaces where they live and spend a good part of their lives. Furthermore, this process of school dropout can restrict participation in sports, games and socializing with friends, important acts for the development of creativity, motor coordination, spatial perception, among other essential skills for full development^(4)^.

The main source of information about menstruation is mothers, followed by sisters, teachers, friends^(17,20,24,25)^ and the media^(32)^. However, the information is transmitted in an inaccurate and incomplete manner^(19,20,36)^, in addition to there being a lack of emotional support during the menstrual period^(20)^. Thus, although family and school are important support settings for people who menstruate, the lack of training, knowledge and tools makes it difficult to approach menstruation in a timely and appropriate manner^(19,25,37,38)^, in addition to reinforcing taboos and stigmas surrounding menstruation.

Furthermore, not talking about menstruation means making a physiological and recurring phenomenon invisible, in addition to feeding myths and taboos that are extremely harmful to women, girls and people who menstruate in general^(4)^.

Due to cultural and religious beliefs, menstruation is not considered an appropriate topic of discussion in Egypt^(32)^. In countries such as Zimbabwe and India, women and girls were excluded from religious rituals because they were considered impure during menstruation^(23,25)^, and were not supposed to cook because it would make men weak^(23)^. Historically, these behaviors have manifested in the perpetuation of taboos and stigmas associated with religious and cultural institutions, which can influence norms related to menstruation and the overall health of menstruating people exposed to these beliefs. Therefore, the act of menstruating can be a stressful event in some societies^(39)^, such as Zimbabwe, India and Egypt^(23,25,32)^, where different religious beliefs impact menstruation and distance women, girls and people who menstruate from health education, communication on the topic and menstrual health care, contributing to period poverty.

These issues prevent people who menstruate from talking about their situation and asking for help when needed, due to the need to hide their periods, causing stress and fear of the risk of menstrual blood exposure and resulting stigma^(17,18,21,22,36)^. Furthermore, women who do not have the money to buy menstrual products have their menstrual care practices severely impaired, causing mental disorders such as anxiety^(22)^ and depression^(33)^.

In addition to the consequences for mental health, the difficulty in accessing water, sanitation, painkillers and menstrual hygiene products directly impacts the lives of people who menstruate, causing loss of productivity^(19,20)^, self-esteem^(23)^, socialization^(22,25,37,38)^ and gender discrimination^(23,25,32)^. Difficulty in proper menstrual management can lead to school absenteeism^(19,20,23,25,28,29,33,36)^, in addition to absence from work^(29)^.

People who menstruate also face monthly hormonal changes that cause a myriad of signs and symptoms, including mood swings, fatigue, pain and bloating, in addition to the practical aspects of the menstrual cycle, such as blood collection and maintaining minimal hygiene to avoid infections^(40)^.

Menstrual health is a comprehensive part of overall health, as menstruation can have a significant impact on the physical, mental and social well-being of people who menstruate, confirming the World Health Organization’s definition that health is a state of complete physical, mental and social well-being, not merely the absence of disease or infirmity.

The normal menstrual cycle brings many changes for girls, women, non-binary individuals and transgender men, which affect their lives in many ways. Unfortunately, few have the means to face them with dignity^(40)^, with consequent improvisation of inadequate materials, such as pieces of cloth^(16-18,20,23,30)^, handkerchiefs^(16,18)^, socks^(20)^, mattress fibers^(17)^, cotton^(18,23,34)^, toilet paper and diapers^(16,36)^, in addition to newspapers, to contain menstruation^(23)^.

Changing pads is prolonged for 12 to 14 hours^(16)^, or use is restricted to just two or three pads over an entire period of three or more days due to the lack of menstrual hygiene products^(21)^ as well as adequate infrastructure to perform menstrual hygiene^(16)^. Improper management of menstruation can cause problems such as allergies and irritation of the skin and mucous membranes, urogenital infections and even a condition known as toxic shock syndrome, which can cause death^(4)^.

Taking care of menstruation is a vital aspect of the health of girls, women, non-binary individuals and transgender men. Experiencing this moment with access to the information and supplies necessary for menstrual hygiene is a right of every person who menstruates. However, gaps such as menstrual concealment are still observed among women and people who menstruate, whether in the social^(22)^, work^(19)^ or academic environment^(17-21,23,25,28,38)^, due to ingrained taboos in society about menstruation^(17-23,25,28,38)^. Invisibility perpetuates unmet menstrual needs, leading to a lack of interventions that promote strategies to combat period poverty.

The lack of adequate resources for hygiene management affects the dignity, health and well-being of people who menstruate, and is a disrespect for human rights and a condition that distances the achievement of seven Sustainable Development Goals (SDGs) such as: SDG 1 - No poverty; SDG 3 - Good health and well-being; SDG 4 - Quality education; SDG 5 - Gender equality; SDG 6 - Clean water and sanitation; SDG 8 - Decent work and economic growth; and SDG 12 - Responsible consumption and production^(4)^.

As a multidimensional and transdisciplinary phenomenon, period poverty requires equally complex coping strategies^(4)^. Improving access to information and safe and secure public toilets^(16,17,35)^, providing continuous menstrual hygiene products^(16,24,33,35)^ and reducing the cost of sanitary pads to increase access for poor families^(32)^ would be strategies to improve menstrual hygiene management and, consequently, the opportunities and development of people who menstruate^(16,17,24)^.

The family and school are also important settings for combating period poverty. To this end, schools must ensure the availability of menstrual hygiene products^(18,19,21,25,28,31,37)^, painkillers^(18,19,25)^, improved WASH facilities^(18,19,21,25-27,37)^, interventions that address knowledge on the topic, and physical and psychosocial aspects of menstruation^(18,19,21,25,26,29,37)^, which would improve the menstrual experience in the academic environment^(18,19,21,25,26,28,29,37)^. There is also a need for schools to create health education programs for families, thus ensuring that myths and misconceptions about menstruation are not passed on, in addition to raising awareness of the importance of family support during this period^(24,32,37)^.

Furthermore, the role of the media and Civil Society Organizations in this process of combating period poverty is fundamental^(19,32,34)^, especially government actions to improve infrastructure and develop interventions on reproductive and menstrual health in schools and in the community^(36)^.

Interventions that promote equal access to education, provide reproductive health education and address the compounding effects of period poverty on the experience of menstruating people can be facilitators to help them achieve their rights, develop their full potential^(17)^ and achieve the SDG targets related to health and well-being, gender equality and the empowerment of all people who menstruate^(4)^.

### Study limitations

This study had limitations regarding the number of databases searched, which may have contributed to limiting access to other data.

### Contributions to nursing, health or public policy

The findings are important for nursing and public health, as they enable the understanding of period poverty as a public health problem and provide support for the formulation of strategies to be implemented and public policies to achieve menstrual dignity as a human right, in addition to contributing to the visibility of the topic in national literature, since a Brazilian publication was found in the section carried out in this review.

## CONCLUSIONS

While lack of financial resources is a major driver of period poverty, other socioeconomic, environmental, and familial factors require urgent policy attention. Social and cultural constructions of menstruation reinforce taboos around menstruation, which materialize in negative experiences for people who menstruate. There is also a need to ensure affordable menstrual products and adequate infrastructure to promote the health of people who menstruate and achieve gender equality. Future research is needed with broader populations of people who menstruate, including transgender and non-binary populations.
