# Supplementary material for: Period poverty: a scoping review
Source: Rev Bras Enferm. 2025 Jun 20;78(2):e20240567. doi: 10.1590/0034-7167-2024-0567 (PMC12185138; doi:10.1590/0034-7167-2024-0567)
Supplement: Translation

# Translation: Pobreza menstrual: revisão de escopo

**DOI:** 10.1590/0034-7167-2024-0567pt

**Authors:** Mayara Cristina de Paula, Bruna Borlina Monteiro, Ludmila de Oliveira Ruela, Mônica Maria de Jesus Silva

**Affiliations:** I Universidade de São Paulo. Ribeirão Preto, São Paulo, Brasil

**Keywords:** Enfermagem, Menstruação, Pobreza, Produtos de Higiene Menstrual, Saúde Sexual e Reprodutiva.

## Abstract

**Objetivos:**

mapear as evidências, perspectivas e lacunas sobre a pobreza menstrual.

**Métodos:**

revisão de escopo, realizada em maio de 2022 nas bases de dados MEDLINE via PubMed, Scopus, LILACS, CINAHL, EMBASE e *Web of Science*, de acordo com as recomendações do JBI.

**Resultados:**

a amostra final foi composta por 23 estudos. Observaram-se quatro fatores preponderantes em relação à pobreza menstrual: fatores associados à pobreza menstrual; impactos da pobreza menstrual; gestão da higiene menstrual; e estratégias de enfrentamento.

**Conclusões:**

a pobreza menstrual é uma questão de saúde pública e direitos humanos, especialmente para pessoas em situação de vulnerabilidade socioeconômica. Apesar do crescente compromisso internacional com o foco na promoção da saúde menstrual, ela ainda não recebe a ênfase adequada. Evidenciou-se a importância da implementação de estratégias de enfrentamento e superação da pobreza menstrual para promoção da saúde menstrual e alcance da igualdade de gênero.

## INTRODUÇÃO

A saúde menstrual e a sua importância têm sido historicamente negligenciadas, principalmente em virtude dos tabus e equívocos em torno da menstruação e dos reflexos do patriarcado no conhecimento e nos sistemas de saúde em todo o mundo^(1)^.

A saúde menstrual necessita ser compreendida como ferramenta de promoção da saúde das pessoas que menstruam, e está vinculada às experiências relacionadas ao ciclo menstrual, o que inclui o combate aos tabus, desinformação, escassez de dados, estigma e discriminação relacionado à menstruação^(2)^.

Ressalta-se que, neste estudo, utilizou-se o termo “pessoas que menstruam” para não excluir pessoas que têm um ciclo menstrual, mas não se identificam como mulheres, e, assim, incluir mulheres, homens transgênero e pessoas não binárias.

A saúde menstrual envolve o alcance das necessidades de higiene menstrual adequadas com o acesso das pessoas que menstruam a informações precisas sobre a menstruação; a produtos menstruais limpos que podem ser trocados em particular quantas vezes forem necessárias; a materiais sanitários, sabão e água para lavagem e limpeza; a um local para descarte higiênico dos produtos utilizados como os absorventes reutilizáveis; e a instalações sanitárias para lavagem limpas e seguras como banheiros^(1,3)^.

No entanto, nem todas as pessoas que menstruam têm acesso a esses recursos, e as desigualdades se fazem presentes em virtude de iniquidades sociais e de saúde, o que as insere em uma situação de pobreza menstrual, que se refere às barreiras financeiras, sociais, culturais e políticas para o acesso a produtos menstruais e educação^(1)^. Entre essas barreiras, incluem-se recursos financeiros para aquisição de suprimentos para a higiene menstrual; transporte para se deslocar aos centros de compra dos suprimentos em grandes quantidades de forma econômica; acesso à saneamento básico e privacidade; proteção e segurança no acesso aos banheiros; e acesso à informação para gerenciar o ciclo menstrual^(3)^.

Essa condição de pobreza menstrual afeta principalmente pessoas em situação de vulnerabilidade, uma vez que ela é fortemente sustentada pelas desigualdades sociais e por tabus que limitam o processo natural que caracteriza a menstruação^(4)^.

Diante desse contexto, em 2014, a Organização das Nações Unidas (ONU) reconheceu o direito das mulheres à higiene menstrual como uma questão de saúde pública e de direitos humanos. Porém, o que deveria ser um direito é, muitas vezes, um luxo. Segundo a ONU Mulheres, 12,8% da população feminina do planeta vive em situação de pobreza menstrual; 526 milhões de pessoas não têm acesso a um banheiro para menstruar com dignidade; e 1,25 bilhões não têm acesso a um banheiro seguro^(5)^.

No Brasil, cerca de 30 milhões de mulheres menstruam no Brasil e gastam, ao longo da vida, de três a nove mil reais com absorventes, e 1,24 milhão de meninas não possuem acesso a papel higiênico nos banheiros das escolas em que estudam^(4,6)^.

Esse cenário de pobreza menstrual promove experiências negativas em torno da menstruação como: o absenteísmo escolar e no trabalho devido à falta de suprimentos de higiene menstrual; a perda de produtividade no período menstrual; a ausência em atividades sociais; a discriminação de gênero^(7)^; a precariedade de condições básicas com consequente improvisação de materiais inadequados, como panos, lenços e/ou toalhas de papel ou o uso prolongado de absorventes internos^(3)^; o desenvolvimento de infecções; e a baixa qualidade de vida relacionada à saúde^(8-9)^.

Tais consequências evidenciam que a superação da pobreza menstrual em todas as suas nuances é fundamental para promoção da saúde das pessoas que menstruam e para o alcance da igualdade de gênero. Para tanto, a disseminação de informação, o acolhimento, a visibilidade em pautas governamentais e a democratização do acesso à insumos menstruais são necessários.

Nesse sentido, a pesquisa, a prática e as políticas que versam sobre a gestão da saúde menstrual e da superação da sua pobreza envolvem um número crescente de atores, como pesquisadores, profissionais, formuladores de políticas, empreendedores sociais e sociedade civil, que globalmente têm trabalhado para abordar os fatores sociais, ambientais e políticos que reforçam os desafios relacionados à menstruação enfrentados por pessoas que menstruam em diversos contextos^(10)^.

No entanto, apesar de a pobreza menstrual ter recebido recentemente essa maior atenção global como uma questão de saúde pública e direitos humanos, especialmente em países com poucos recursos^(11,12)^, e do crescente compromisso internacional com o foco na promoção da saúde menstrual, as mesmas ainda não recebem a ênfase adequada e ainda há muito a ser feito.

Desse modo, conhecer as lacunas e perspectivas relativas à pobreza menstrual é fundamental para traçar estratégias e implementar ações para a sua superação e para a promoção da saúde das pessoas que menstruam.

## OBJETIVOS

Mapear as evidências, perspectivas e lacunas sobre a pobreza menstrual.

## MÉTODOS

### Aspectos éticos

Não foi necessária a apreciação ética, visto que o material utilizado é de domínio público e não envolve seres humanos.

### Desenho do estudo

Trata-se de revisão de escopo da literatura, na qual foram seguidas as diretrizes propostas pelo JBI^(13)^. Adicionalmente, o *Preferred Reporting Items for Systematic Reviews and Meta-Analyses Extension for Scoping Reviews* (PRISMA-ScR) foi utilizado para a redação do estudo^(14)^.

### Período e local do estudo

Foram utilizadas as bases de dados MEDLINE (via PubMed), CINAHL (via EBSCO), *Web of Science,* Scopus, LILACS e EMBASE.

Os descritores foram selecionados nas bases de dados em março e abril de 2022. O levantamento de dados ocorreu em maio de 2022.

### Critérios de inclusão e exclusão

Foram considerados elegíveis os estudos que respondessem à pergunta de pesquisa, atendessem ao objetivo do estudo, com abordagem temática sobre a pobreza menstrual. Não houve restrição de idioma e temporal. Foram excluídos estudos em formato de comentário, editorial, protocolo/revisão, debate, estudo de caso, estudo teórico/reflexão, relatório técnico científico, crítica, análise de livro/filme, resumo de congresso/evento científico.

### Protocolo do estudo

Para orientar a revisão, utilizou-se a combinação mnemônica PCC^(13)^, composta por População (não se aplica), Conceito (menstruação) e Contexto (pobreza menstrual). Ressalta-se que a população não foi restrita a mulheres e meninas, para não limitar os estudos quanto ao gênero e visando abranger todas as pessoas com útero que menstruam. Desta forma, elaborou-se a questão de pesquisa: quais as evidências sobre a situação de pobreza menstrual disponíveis na literatura?

A seguir, iniciou-se a busca pelos descritores que representam o objeto de estudo na MEDLINE (via PubMed) e CINAHL (via EBSCO), seguida de uma pesquisa mais ampla, utilizando as mesmas palavras-chave e termos de pesquisa nas bases de dados *Web of Science,* Scopus, LILACS e EMBASE.

Posteriormente, foram identificados os descritores no *Medical Subject Headings* (MeSH), para descritores em inglês, e nos Descritores em Ciências da Saúde (DeCS), para descritores em português e espanhol. Os descritores selecionados foram *“Menstruation*”, “*Menstrual Hygiene Products*”, “Menstrual*”, “Poverty” e *“Period Poverty*” nos idiomas inglês, espanhol e português. A estratégia para a busca foi desenvolvida em conjunto com profissional bibliotecário de uma universidade pública do estado de São Paulo, adicionando os operadores booleanos *AND* e *OR* aos descritores.

Após a determinação dos descritores e a criação da estratégia acima, procedeu-se à realização das buscas em cada base de dados. Em seguida, procedeu-se à busca externa no *Google Scholar* ^®^, visando à literatura cinzenta, como preconizado pelo JBI^(13)^. Por fim, a lista de referências dos principais artigos incluídos foi verificada para recuperar artigos pertinentes.

A busca dos estudos foi realizada por dois revisores de maneira independente e cega. Primeiramente, realizou-se a leitura dos títulos, resumos e descritores. Posteriormente, realizou-se a leitura dos textos completos. Em cada fase, foi realizado consenso entre as revisoras por meio de discussão. Em caso de discordância, um terceiro revisor poderia ser acrescentado, o que não foi necessário.

Para auxiliar na seleção e no gerenciamento, os estudos foram exportados para o *software* Rayyan QCIR^®(15)^.

### Análise dos resultados

Os dados foram extraídos, organizados e caracterizados em uma adaptação de um formulário recomendado pelo JBI, contendo dados de publicação (ano, autores e país de publicação), objetivos do estudo, características metodológicas (população de estudo) e principais resultados (desfechos, achados ou contribuições).

As informações extraídas foram organizadas em uma planilha eletrônica no *Microsoft Office Excel* ^®^. Os dados de caracterização dos estudos foram analisados por estatística descritiva simples. As evidências sobre a pobreza menstrual foram interpretadas por meio de consenso realizado pelos pesquisadores após extração dos resultados de forma independente.

## RESULTADOS

Dos 5.246 estudos identificados na busca inicial, 23 foram incluídos na amostra final. A Figura 1 especifica os resultados das etapas da análise, seguindo o modelo PRISMA *Flow Diagram* ^(14)^.

**Figura 1:** Fluxograma de seleção dos estudos, Ribeirão Preto, São Paulo, Brasil, 2022

*Fonte: adaptado de PRISMA-ScR^(13)^.*

*Legenda: razão 1: não responde à pergunta; razão 2: não disponível em texto completo; razão 3: comentário/editorial; razão 4: protocolo/revisão; razão 5: debate; razão 6: estudo de caso; razão 7: estudo teórico/reflexão; razão 8: dissertação; razão 9: relatório técnico científico; razão 10: crítica; razão 11: análise de livro; razão 12: resumo de congresso.*

O Quadro 1 apresenta a síntese dos resultados.

**Quadro 1:** Caracterização dos estudos quanto ao título, ano, país, delineamento metodológico, periódico de publicação e resultados sobre a pobreza menstrual, Ribeirão Preto, São Paulo, Brasil, 2022

ID	Título/ delineamento metodológico	País/ ano/ periódico	Resultados sobre a pobreza menstrual
1^(16)^	*Unmet Menstrual Hygiene Needs Among Low-Income Women* Estudo transversal	Estados Unidos, 2019 *Obstetrics & Gynecology*	- Falta de acesso a produtos de higiene menstrual e segurança para o uso de instalações sanitárias; - Desafios para mulheres de baixa renda semelhantes aos vividos em países com menos recursos que os Estados Unidos da América.
2^(17)^	*Adolescent Experience of Menstruation in Rural Kenya* Estudo qualitativo	Quênia, 2016 *Nursing Research*	- Fontes de informação sobre menstruação foram escola, família e colegas; - Uso de materiais alternativos inadequados pela falta de absorventes; - Absenteísmo escolar e impacto na educação; - Menstruação relacionada a sentimentos ruins.
3^(18)^	*Menstrual health and school absenteeism among adolescent girls in Uganda (MENISCUS): a feasibility study* Estudo misto	Uganda, 2018 *BMC Women’s Health*	- Fonte de informação sobre menstruação foram as mães; - Normas culturais desfavoráveis à menstruação; - Uso de materiais alternativos inadequados pela falta de absorventes; - Absenteísmo escolar.
4^(19)^	*Menstrual hygiene management amongst schoolgirls in the Rukungiri district of Uganda and the impact on their education: a cross-sectional study* Estudo misto	Uganda, 2014 *Pan African* *Medical Journal*	- Absenteísmo escolar; - Falta de acesso a absorventes; - Más condições dos banheiros nas escolas para manejo da higiene menstrual.
5^(20)^	*Emotional and Psychosocial Aspects of Menstrual Poverty in Resource-Poor Settings: A Qualitative Study of the Experiences of Adolescent Girls in an Informal Settlement in Nairobi* Estudo qualitativo	Quênia, 2013 *Health Care for Women International*	- Fonte de informação sobre menstruação era família, amigos e professores. Informações limitadas; - Menstruação relacionada a sentimentos ruins; - Menstruação afetava concentração e frequência nas aulas.
6^(21)^	*Physical, Social, and Political Inequities Constraining Girls’ Menstrual Management at Schools in Informal Settlements of Nairobi, Kenya* Estudo qualitativo	Quênia, 2017 *Journal Urban Health*	- Falta de acesso à informação sobre menstruação; - Distribuição gratuita de absorventes nas escolas; - Vergonha do sangue menstrual.
7^(22)^	*‘Period poverty’ in Stoke-on-Trent, UK: New insights into gendered poverty and the lived experiences of austerity* Estudo qualitativo	Inglaterra, 2020 *Journal of Poverty and Social Justice*	- “Pobreza de período” relacionada à baixa renda; - Exclusão social devido à menstruação; - Impactos na saúde mental.
8^(23)^	*Menstrual hygiene - A salient hazard in rural schools: A case of Masvingo district of Zimbabwe* Estudo qualitativo	Zimbábue, 2016 *Jàmbá: Journal of Disaster Risk*	- Crenças religiosas e culturais desfavoráveis á menstruação; - Uso de materiais alternativos inadequados pela falta de absorventes; - Limitações para o manejo da higiene menstrual; - Absenteísmo e baixo desempenho escolar.
9^(24)^	*Menstrual hygiene management practice among adolescent girls: an urban-rural comparative study in Rajshahi division, Bangladesh* Estudo transversal	Índia, 2022 *BMC Women’s Health*	- Fonte de informação sobre a menstruação eram as mães, amigas e professoras; - Dificuldade de acesso a absorventes; - Fatores socioeconômicos relacionados a boas práticas de higiene menstrual.
10^(25)^	*Effect of menstruation on girls and their schooling, and facilitators of menstrual hygiene management in schools: surveys in government schools in three states in India, 2015* Estudo transversal	Índia, 2019 *Journal of Global Health*	- Crenças religiosas e culturais desfavoráveis à menstruação; - Sentimentos ruins relacionados à menstruação; - Falta de condições para manejo de higiene menstrual; - Facilitadores para reduzir absenteísmo escolar durante a menstruação.
11^(26)^	*Secrets, shame and discipline: School girls’ experiences of sanitation and menstrual hygiene management in a peri-urban community in Ghana* Estudo quantitativo	Gana, 2019 Taylor & Francis	- Falta de condições para o manejo da higiene menstrual; - Informações dadas após a menarca mais focadas na saúde sexual e reprodutiva do que na higiene menstrual.
12^(27)^	*Menstrual hygiene management among girls at a peri-urban senior high school in the Volta Region, Ghana* Estudo descritivo	Gana, 2020 *African Journal of Midwifery and Women’s Health*	- Menstruação vista como anti-higiênica; - Falta de informação e de acesso a absorventes; - Absenteísmo escolar; - Falta de condições para manejo da higiene menstrual nas escolas.
13^(28)^	*Menstruation Management in United States Schools and Implications for Attendance, Academic Performance, and Health* Estudo qualitativo	Estados Unidos, 2019 *Women’s Reproductive* *Health*	- Menstruação como algo negativo; - Falta de absorvente atrapalha na concentração nas aulas; - Distribuição de absorventes gratuitos na escola reduz chance de absenteísmo e de impacto na aprendizagem.
14^(29)^	*Menstruation practice among school and out-of-school adolescent girls, Lao PDR* Estudo descritivo	Laos, 2020 *Global Health Action*	- Absenteísmo escolar e laboral devido à menstruação; - A menstruação era mais discutida com mães e pouco discutida com pais; - Fatores relacionados à boa prática de manejo da higiene menstrual.
15^(30)^	*Menstrual hygiene management among women and adolescent girls in the aftermath of the earthquake in Nepal* Estudo misto	Nepal, 2018 *BMC Women’s Health*	- Higiene menstrual foi considerada a sexta maior necessidade após o terremoto no Nepal; - Nenhuma mulher recebeu doação de absorventes; - Condições ruins par manejo da higiene menstrual; - Uso de materiais alternativos não indicados na falta de absorventes.
16^(31)^	*Empowering Women: Teaching Ethiopian Girls to Make Reusable Sanitary Pads* Estudo transversal	Etiópia, 2014 *Clinical Scholars Review*	- O projeto ensinava sobre empoderamento, menstruação e a costurar absorventes reutilizáveis; - Fala de acesso a absorventes e vergonha do sangue menstrual; - Más condições dos banheiros para manejo da higiene menstrual; - O uso dos absorventes reutilizáveis teve impacto positivo.
17^(32)^	*Menstrual Hygiene among Adolescent Schoolgirls in Mansoura, Egypt* Estudo transversal	Egito, 2005 Taylor & Francis	- Fatores socioeconômicos relacionados à educação menstrual e acesso a absorventes; - Falta de informação sobre menstruação; - Fontes de informação foram as mídias e mães; - Crenças culturais e religiosas desfavoráveis à menstruação.
18^(33)^	*Period poverty and mental health implications among college-aged women in the United States* Estudo transversal	Estados Unidos, 2021 *BMC* *Women’s Health*	- Fatores relacionados ao acesso a absorventes; - Alternativas para lidar com a falta de absorventes; - Associação entre ter vivenciado a pobreza menstrual e ter sintomas de depressão.
19^(34)^	*Period poverty: menstrual health hygiene issues among adolescent and young Venezuelan migrant women at the northwestern border of Brazil* Estudo transversal	Brasil, 2021 *Reproductive* *Health*	- Entre os jovens migrantes venezuelanos em Boa Vista, Brasil, havia falta de acesso a produtos adequados de higiene menstrual, condições sanitárias e banheiros; - Menstruação vista como um tabu; - Falta de informação sobre menstruação; - Menstruação estava associada a sentimentos negativos.
20^(35)^	*Understanding period poverty: Socio-economic inequalities in menstrual hygiene management in eight low-and middle-income countries* Estudo transversal	África do Sul, 2021 *International Journal of Environmental Research and* *Public Health*	- Falta de acesso a banheiros em condições para o manejo da higiene menstrual e a absorventes; - Fatores relacionados à falta de insumos para a higiene menstrual.
21^(36)^	*Factors impacting on menstrual hygiene and their implications for health promotion* Estudo qualitativo	Zâmbia, 2018 *Global Health Promotion*	- Estigma e cultura desfavoráveis à menstruação; - Falta de informação sobre menstruação; - Absenteísmo e desistência escolar; - Uso de materiais alternativos pela falta de absorventes.
22^(37)^	*Menstrual hygiene management in rural schools of Zambia: a descriptive study of knowledge, experiences and challenges faced by schoolgirls* Estudo quantitativo	Zâmbia, 2019 *BMC Public Health*	- Falta de informação sobre gestão de higiene menstrual; - Experiências negativas na primeira menstruação; - Falta de acesso a absorventes; - Absenteísmo escolar; - Falta de condições sanitárias para manejo da higiene menstrual na escola.
23^(38)^	*Desafíos de la menstruación en niñas y adolescentes de comunidades rurales del pacífico colombiano* Estudo misto	Colômbia, 2017 *Rev. Salud Pública*	- Falta de condições para o manejo de higiene menstrual na escola; - Absenteísmo escolar; - Falta de informação sobre menstruação e a menstruação como uma experiência negativa; - Não haviam políticas públicas para educação menstrual.

Os estudos foram publicados entre 2005 e 2022, sendo um (4,3%) publicado em 2005^(32)^, um (4,3%), em 2013^(20)^, dois (8,7%), em 2014^(19,31)^, dois (8,7%), em 2016^(19,23)^, dois (8,7%), em 2017^(21,38)^, três (13%), em 2018^(18,30,36)^, cinco (21,7%), em 2019^(16,18,26,28,37)^, três (13%), em 2020^(22,27,29)^, três (13%), em 2021^(33-35)^, e um (4,3%), em 2022^(24)^.

Os países com maior número de publicações foram Estados Unidos^(16,28,33)^ e Quênia^(17,20,21)^, com três estudos cada (26%); Índia^(23,25)^, Uganda^(18,19)^, Gana^(26,27)^ e Zâmbia^(36,37)^, com dois artigos cada (34,8%); seguidos de Brasil^(37)^, Inglaterra^(22)^, Zimbábue^(23)^, Laos^(29)^, Nepal^(30)^, Etiópia^(31)^, Egito^(32)^, África do Sul^(35)^ e Colômbia^(34)^, que apresentaram um estudo cada (38,7%).

Em relação à metodologia, nove (39,1%) eram estudos qualitativos^(17,20-23,26,28,36,37)^, oito (34,7%), transversais^(16,22,24,25,31,32,37)^, quatro (17,5%), mistos^(18,19,30,38)^, e dois (8,7%), descritivos^(27,29)^.

Os resultados evidenciaram quatro fatores preponderantes entre as perspectivas e lacunas em relação à pobreza menstrual: fatores associados à pobreza menstrual; impactos da pobreza menstrual; gestão da higiene menstrual; e estratégias de enfrentamento.

## DISCUSSÃO

Como destaca o relatório do Fundo das Nações Unidas para a Infância, a pobreza menstrual se caracteriza como um fenômeno transdisciplinar e multidimensional vivenciado por pessoas que menstruam devido à presença de barreiras econômicas, sociais, culturais e políticas para o gerenciamento da higiene menstrual^(4)^, assim como observado nos estudos desta revisão^(16-38)^.

Alguns fatores socioeconômicos relacionam-se à pobreza menstrual, como baixa renda^(16,17,22,35)^, escolaridade^(24,29,35)^ e habitação em zona rural^(17,35)^. Diante dos fatores econômicos, pessoas que menstruam de baixa renda eram mais propensas a ter dificuldade no acesso a produtos de higiene menstrual e no acesso a saneamento básico nas escolas e residências^(16,17,36)^.

Além disso, pessoas que menstruam com residência em áreas rurais são frequentemente forçadas a faltar à escola^(17,36)^, em decorrência dos desafios que enfrentam para o gerenciamento da higiene menstrual^(37)^, como a falta de acesso a absorventes e infraestrutura adequada^(17)^.

Nesse sentido, embora a pobreza menstrual seja uma experiência global, o impacto negativo na qualidade de vida e bem-estar pode ser mais pronunciado para pessoas que vivem em condições de pobreza e situação de vulnerabilidade^(4)^.

Em contrapartida, alguns fatores se associam à garantia de boas práticas para higiene menstrual, como residir em área urbana, integrar família pequena^(24)^, escolaridade adequada dos pais^(24,29)^, e conversas entre mãe e filha^(29)^. Apesar de ainda haver associação entre acesso a produtos de higiene menstrual com baixa renda e residência em zona rural^(16,17,22,35)^, viver em áreas urbanas, ao ser analisado conjuntamente com renda, tem influência para maior ou menor pobreza menstrual, dependendo do país em questão^(35)^.

A ausência de produtos para o gerenciamento da saúde menstrual pode afetar mesmo as pessoas que não estão em situação de pobreza. Tal fato pode ser entendido devido ao absorvente ser considerado um produto supérfluo, sobrando pouca ou nenhuma renda para ser utilizada para compra de insumos que ajudem a garantir a dignidade menstrual^(4)^.

Mesmo a falta de recurso financeiro sendo um dos principais determinantes pela situação de pobreza menstrual^(16,17,22,35)^, pessoas que menstruam em países de baixa renda enfrentam desafios de higiene menstrual semelhantes aos de pessoas em países com mais recursos^(16)^, evidenciando que a pobreza menstrual é um fenômeno global que necessita ser analisado holisticamente.

Tabus, restrições de informações e estigma em torno da menstruação, bem como a falta de recursos adequados para o gerenciamento da higiene menstrual, afetam negativamente as habilidades de pessoas que menstruam de socializar e participar da escola^(17-20,23,25,28,29,31,33)^. Barreiras sociais e físicas incluem banheiros, água e descarte inadequados dentro das dependências da escola^(18-21,23,25-27,29,34,35,37,38)^, falta de acesso a analgésicos^(18,21,32)^, orientação e apoio insuficientes para administrar seus períodos menstruais^(18,20,21,32,37,38)^, e dificuldade de acesso aos produtos menstruais^(17-20,26,27,35)^.

As principais razões apontadas para o porquê de a menstruação manter as pessoas que menstruam afastadas da escola incluem: dor^(18,19,37)^, pois algumas não tomavam analgésicos por falta de conhecimento ou falta de dinheiro^(20)^; medo de humilhação, devido à exposição do sangue menstrual^(17-20,25,37,38)^; falta de absorventes^(17-19,38)^; falta de privacidade; e instalações inadequadas para o gerenciamento da higiene menstrual^(19,21,37)^.

Os dados apresentados demonstram como crianças e adolescentes que menstruam têm seus direitos à escola de qualidade, saúde, incluindo a saúde sexual e reprodutiva violados quando seus direitos à água, saneamento e higiene não são garantidos nos espaços em que convivem e passam boa parte de suas vidas. Além do mais, esse processo de evasão escolar pode restringir a participação em esportes, brincadeiras e a convivência com amigos, atos importantes para o desenvolvimento da criatividade, coordenação motora, percepção espacial, entre outras competências essenciais para o desenvolvimento pleno^(4)^.

A principal fonte de informação sobre a menstruação reside nas mães, seguidas de irmãs, professores, amigas^(17,20,24,25)^ e mídia^(32)^. Entretanto, as informações são transmitidas de maneira imprecisa e incompleta^(19,20,36)^, além de haver falta de apoio emocional durante o período menstrual^(20)^. Assim, apesar de a família e a escola serem importantes cenários de apoio para pessoas que menstruam, a falta de capacitação, conhecimento e ferramentas dificulta a abordagem da menstruação de forma oportuna e adequada^(19,25,37,38)^, além de reforçar tabus e estigmas em torno da menstruação.

Ademais, não falar sobre a menstruação significa tornar invisível um fenômeno fisiológico e recorrente, além de alimentar mitos e tabus extremamente danosos às mulheres, meninas e pessoas que menstruam de maneira geral^(4)^.

Devido às crenças culturais e religiosas, a menstruação não é considerada um tópico apropriado de discussão no Egito^(32)^. Em países como Zimbábue e Índia, mulheres e meninas eram excluídas dos rituais religiosos porque eram consideradas impuras durante a menstruação^(23,25)^, e não deveriam cozinhar porque tornaria os homens fracos^(23)^. Historicamente, esses comportamentos têm se manifestado com a perpetuação de tabus e estigmas associados a instituições religiosas e culturais, o que pode influenciar normas relacionadas à menstruação e a saúde geral de pessoas que menstruam expostas a essas crenças. Logo, o ato de menstruar pode ser um evento desgastante em algumas sociedades^(39)^, como Zimbábue, Índia e Egito^(23,25,32)^, onde as diferentes crenças religiosas impactam a menstruação e distanciam mulheres, meninas e pessoas que menstruam da educação em saúde, da comunicação sobre o tema e dos cuidados em saúde menstrual, contribuindo para a pobreza menstrual.

Essas questões impedem as pessoas que menstruam de falar sobre sua situação e pedir ajuda, quando necessário, devido à necessidade de ocultação da menstruação, causando estresse e medo do risco de exposição do sangue menstrual e estigma resultante^(17,18,21,22,36)^. Outrossim, mulheres que não têm dinheiro para comprar produtos menstruais têm suas práticas de cuidados menstruais severamente prejudicadas, ocasionando distúrbios mentais como ansiedade^(22)^ e depressão^(33)^.

Além das consequências para a saúde mental, a dificuldade de acesso a serviços de água, saneamento, analgésicos e produtos de higiene menstrual impacta diretamente a vida de pessoas que menstruam, ocasionando a perda de produtividade^(19,20)^, autoestima^(23)^, socialização^(22,25,37,38)^ e discriminação de gênero^(23,25,32)^. A dificuldade da gestão menstrual adequada pode levar ao absenteísmo escolar^(19,20,23,25,28,29,33,36)^, além da falta ao trabalho^(29)^.

As pessoas que menstruam também enfrentam todo mês as alterações hormonais responsáveis por uma infinidade de sinais e sintomas, incluindo alterações de humor, fadiga, dores e inchaço, somados aos aspectos práticos do ciclo menstrual, como a coleta de sangue e a manutenção de higiene mínima, para evitar infecções^(40)^.

A saúde menstrual é parte integrante da saúde geral, pois a menstruação pode ter um impacto significativo no bem-estar físico, mental e social de pessoas que menstruam, ratificando a definição da Organização Mundial da Saúde de que a saúde é um estado de completo bem-estar físico, mental e social, não apenas a ausência de doença ou enfermidade.

O ciclo menstrual normal traz inúmeras mudanças para meninas, mulheres, indivíduos não binários e homens transgênero, o que afeta suas vidas de várias maneiras. Infelizmente, poucos têm meios para enfrentá-las com dignidade^(40)^, com consequente improvisação de materiais inadequados, como pedaços de pano^(16-18,20,23,30)^, lenços^(16,18)^, meias^(20)^, fibras de colchão^(17)^, algodão^(18,23,34)^, papel higiênico e fraldas^(16,36)^, além de jornais, para conter a menstruação^(23)^.

A troca de absorventes é prolongada por 12 a 14 horas^(16)^, ou o uso restringe-se a apenas dois ou três absorventes durante um período inteiro de três ou mais dias devido à falta de produtos de higiene menstrual^(21)^, assim como de infraestrutura adequada para realizar a higiene menstrual^(16)^. O manejo inadequado da menstruação pode gerar desde problemas como alergia e irritação na pele e mucosas, infecções urogenitais até uma condição conhecida como síndrome do choque tóxico, que pode causar a morte^(4)^.

Cuidar da menstruação é um aspecto vital para saúde de meninas, mulheres, indivíduos não binários e homens transgêneros. Viver esse momento com acesso às informações e aos insumos necessários para higiene menstrual é um direito de toda pessoa que menstrua. No entanto, ainda observam-se lacunas como a ocultação menstrual entre mulheres e pessoas que menstruam, seja no ambiente social^(22)^, de trabalho^(19)^ ou acadêmico^(17-21,23,25,28,38)^, devido a tabus arraigados na sociedade sobre a menstruação^(17-23,25,28,38)^. Na invisibilidade, as necessidades menstruais não atendidas se perpetuam, ocasionando falta de intervenções que promovam estratégias de combate à pobreza menstrual.

A falta de recursos adequados para o gerenciamento da higiene afeta a dignidade, a saúde e o bem-estar de pessoas que menstruam, sendo um desrespeito aos direitos humanos e uma condição que distância o alcance de sete Objetivos de Desenvolvimento Sustentável (ODS) como: ODS 1 - erradicação da pobreza; ODS 3 - Saúde e bem-estar; ODS 4 - Educação de qualidade; ODS 5 - Igualdade de gênero; ODS 6 - Água potável e saneamento; ODS 8 - Trabalho decente e crescimento econômico; e ODS 12 - Consumo e produção responsável^(4)^.

Enquanto fenômeno multidimensional e transdisciplinar, a pobreza menstrual exige estratégias de enfrentamento igualmente complexas^(4)^. Melhorar o acesso à informação e a banheiros públicos seguros e protegidos^(16,17,35)^, fornecer continuamente produtos de higiene menstrual^(16,24,33,35)^ e reduzir os custos de absorventes para aumentar o acesso às famílias pobres^(32)^ seriam estratégias para melhorar o manejo da higiene menstrual e, consequentemente, as oportunidades e o desenvolvimento de pessoas que menstruam^(16,17,24)^.

A família e a escola também se apresentam como importantes cenários de combate à pobreza menstrual. Para isso, é necessário que as escolas assegurem a disponibilidade de produtos de higiene menstrual^(18,19,21,25,28,31,37)^, analgésicos^(18,19,25)^, instalações *WASH* melhoradas^(18,19,21,25-27,37)^, intervenções que abordem o conhecimento sobre a temática, e aspectos físicos e psicossociais da menstruação^(18,19,21,25,26,29,37)^, o que melhorariam a experiência menstrual no ambiente acadêmico^(18,19,21,25,26,28,29,37)^. Há também a necessidade de escolas criarem programas de educação em saúde para as famílias, garantindo, assim, que mitos e crenças erradas sobre a menstruação não sejam repassados, além da conscientização da importância do apoio familiar nesse período^(24,32,37)^.

Além disso, o papel da mídia e de Organizações da Sociedade Civil nesse processo de combate à pobreza menstrual é fundamental^(19,32,34)^, principalmente ações do governo para melhoria da infraestrutura e desenvolvimento de intervenções sobre saúde reprodutiva e menstrual em escolas e na comunidade^(36)^.

Intervenções que promovam a igualdade de acesso à educação, forneçam educação em saúde reprodutiva e que abordem os efeitos potencializadores da pobreza menstrual na experiência de pessoas que menstruam podem ser facilitadores para ajudá-las a atingir seus direitos, desenvolver todo o seu potencial^(17)^ e alcançar as metas dos ODS relacionadas à saúde e bem-estar, à igualdade de gênero e empoderamento de todas pessoas que menstruam^(4)^.

### Limitações do estudo

Este estudo apresentou limitações no que diz respeito ao número de bases de dados pesquisadas, o que pode ter contribuído para limitar o acesso a outros dados.

### Contribuições para as áreas da enfermagem, saúde ou políticas públicas

Os achados são importantes para a enfermagem e para a saúde pública, pois possibilitam a compreensão da pobreza menstrual como um problema de saúde pública e fornecem subsídios para a formulação de estratégias a serem implementadas e de políticas públicas para o alcance da dignidade menstrual como um direto humano, além de contribuírem para a visibilidade da temática na literatura nacional, visto que foi encontrada publicação brasileira no recorte realizado nesta revisão.

## CONCLUSÕES

Embora a falta de recurso financeiro seja um dos principais impulsionadores da pobreza menstrual, outros fatores socioeconômicos, ambientais e familiares exigem atenção política urgente. As construções sociais e culturais sobre a menstruação reforçam tabus em torno da menstruação, que se materializam em experiências negativas para pessoas que menstruam. Destaca-se ainda a necessidade de garantia a produtos menstruais acessíveis e infraestrutura adequada para promover a saúde das pessoas que menstruam e para o alcance da igualdade de gênero. Pesquisas futuras são necessárias com populações mais amplas de pessoas que menstruam, incluindo pessoas transgêneros e populações não binárias.
